# Predictive model of recurrent ischemic stroke: model development from real-world data

**DOI:** 10.3389/fneur.2023.1118711

**Published:** 2023-04-28

**Authors:** Marwa Elsaeed Elhefnawy, Siti Maisharah Sheikh Ghadzi, Orwa Albitar, Balamurugan Tangiisuran, Hadzliana Zainal, Irene Looi, Norsima Nazifah Sidek, Zariah Abdul Aziz, Sabariah Noor Harun

**Affiliations:** ^1^School of Pharmaceutical Sciences, Universiti Sains Malaysia, Penang, Malaysia; ^2^Clinical Research Center, Hospital Seberang Jaya, Penang, Malaysia; ^3^Clinical Research Centre, Hospital Sultanah Nur Zahirah, Terengganu, Malaysia

**Keywords:** recurrent, ischemic stroke, pharmacometrics, time to event model, NONMEM

## Abstract

**Background:**

There are established correlations between risk factors and ischemic stroke (IS) recurrence; however, does the hazard of recurrent IS change over time? What is the predicted baseline hazard of recurrent IS if there is no influence of variable predictors? This study aimed to quantify the hazard of recurrent IS when the variable predictors were set to zero and quantify the secondary prevention influence on the hazard of recurrent ischemic stroke.

**Methods:**

In the population cohort involved in this study, data were extracted from 7,697 patients with a history of first IS attack registered with the National Neurology Registry of Malaysia from 2009 to 2016. A time-to-recurrent IS model was developed using NONMEM version 7.5. Three baseline hazard models were fitted into the data. The best model was selected using maximum likelihood estimation, clinical plausibility, and visual predictive checks.

**Results:**

Within the maximum 7.37 years of follow-up, 333 (4.32%) patients had at least one incident of recurrent IS. The data were well described by the Gompertz hazard model. Within the first 6 months after the index IS, the hazard of recurrent IS was predicted to be 0.238, and 6 months after the index attack, it reduced to 0.001. The presence of typical risk factors such as hyperlipidemia [HR, 2.22 (95%CI: 1.81–2.72)], hypertension [HR, 2.03 (95%CI: 1.52–2.71)], and ischemic heart disease [HR, 2.10 (95%CI: 1.64–2.69)] accelerated the hazard of recurrent IS, but receiving antiplatelets (APLTs) upon stroke decreased this hazard [HR, 0.59 (95%CI: 0.79–0.44)].

**Conclusion:**

The hazard of recurrent IS magnitude differs during different time intervals based on the concomitant risk factors and secondary prevention.

## Introduction

Stroke is the world's second leading cause of death and mortality (1–4). The risk of recurring strokes is much greater for survivors of acute ischemic stroke (IS). For survivors of acute ischemic stroke (IS), the risk of repeated strokes is significantly larger (5–7). In Malaysia, ~33% of the IS population had recurrent stroke (8). In recurrence stroke, neurological damage is usually severe, harder to deal with, and has a higher mortality rate compared with the first stroke (9). Therefore, secondary prevention is crucial to reduce recurrent IS events (9).

The prognosis of recurrent IS has been widely studied. The probability of recurrent IS after the index attack was predicted to vary over time, i.e., it was predicted to range from 11.2% to 30% within the first 24 months (10, 11) and be 9.5% within 5 years after the IS attack (12). In contrast, the most recent study reported that the rate of recurrent IS was 1.2% in the first 30 days, 3.4% within 90 days, 7.4% within 1 year, and 19.4% within 5 years (13). Moreover, the reported risk factors of recurrent stroke vary (14–16), in which hypertension (HTN), atrial fibrillation (AF), diabetes mellitus (DM), hyperlipidemia (HPLD), ischemic heart disease (IHD), and smoking were the most common reported predictors of recurrent stroke (17, 18). Despite improvements in recurrent IS risk classification and prevention measures in the past decades, IS remains a devastating disease. Currently, most of the methods of secondary prevention of IS are focused on reducing and controlling the risk factors that lead to recurrent IS. Nevertheless, does the hazard of recurrent IS change over time?

The Essen Stroke Risk Score (ESRS) is a score that is used to predict stroke recurrence in a hospital-based follow-up study. It includes 9 points depending on risk factors: 2 point for age >75, but only 1 point for 65–75, HTN, DM, previous myocardial infarction, other cardiovascular diseases, peripheral arterial disease, smoking history, and previous TIA or IS (19). The Recurrence Risk Estimator at 90 days (RRE-90) is a web-based prognostic scoring tool designed to calculate 90-day recurrent stroke risk by including risk factors of stroke, such as the history of a mini-stroke or transient ischemic attack (TIA), age, and the type of first stroke the person experienced (20). These conventional recurrent IS prediction scores did not incorporate time to follow the longitudinal natural changes of recurrent IS.

The majority of the previous prognosis studies of recurrent stroke used the most common semi-parametric survival analysis method, i.e., the Cox regression analysis. The Cox model incorporates the effect of covariates on the hazard without quantifying the shape or form of the recurrent stroke hazard rate at baseline. The hazard of the event at baseline is defined as the hazard of having an interest event when all the predictor variables were set to zero or their reference level was set for categorical variables. Thus, in addition to quantifying the effect of predictor variables on the occurrence of the event (e.g., recurrent IS), defining specific shape or distribution of the event hazard at baseline (e.g., just after the index stroke) may allow better prediction of the event of interest, taking into account the natural effect of the disease itself. Studies using this approach on recurrent strokes are still lacking (21, 22).

In this study, we performed a non-conventional way of developing a predictive model for recurrent IS using the parametric approach of the time-to-event analysis. We quantified the specific trend of recurrent IS after the index IS when all the predictor variables were set to zero. This permits more time-dependent prognostic information that better reflects the disease's expected “natural effect.” Moreover, the validated prognostic models of recurrent IS are limited. This study used real-world population-based data of the IS population and aimed to quantify the hazard of recurrent IS when the variable predictors were set to zero, to quantify the hazard of the recurrent IS at different time points after the index IS, and to quantify the secondary prevention influence on the hazard of recurrent ischemic stroke.

## Method

### Patients and data acquisition

This population cohort study used the secondary analysis of data from the National Neurology Registry (NNEUR) of Malaysia. Data of all Malaysian patients with a history of index IS from August 2009 to December 2016 were extracted from the NNEUR of Malaysia. The details on the National Stroke Registry of Malaysia were published previously (23–25). The stroke was diagnosed according to the World Health Organization's criteria (26). All diagnoses were confirmed using brain computed tomography or magnetic resonance imaging. Index IS was defined as the first stroke registered in the NNEUR for patients from 2009 to 2016. Recurrent IS was defined as any IS event recorded by involving hospitals after the index IS for a specific patient in the NNEUR database. Malaysian adults aged above 18 years with a history of IS and registered with NNEUR were included. Non-Malaysian citizens and those with diagnoses other than IS were excluded from the study. The minimum events needed to develop this prognostic model were calculated as 228. Sample size—Survival analysis|Sample Size Calculators (sample-size.net).

### Stroke registry in Malaysia

The NNEUR in Malaysia was established in 2009. It has recorded data from multiethnic stroke cases from 13 states in the country. The NNEUR aims to provide comprehensive epidemiological data on the country's stroke statistics, trends, and management, representing a multicenter, hospital-based registry. The registry development is funded by the Ministry of Health, Malaysia (MOH). A comprehensive explanation of the NNEUR has been previously published (27).

### Ethics approval

Ethical approval for this study was obtained from the Medical Research and Ethics Committee (MREC), Ministry of Health, Malaysia (Research ID: NMRR-08-1631-3189).

### Collected variables

Based on demographic data and concomitant diseases, including DM, HTN, HPLD, IHD, and hyperuricemia, medications used for secondary prevention were tested. They were defined either by physician diagnosis, by patients' electronic records, or from the medication history, and the medications were prescribed during discharge.

### Analysis

The time to the recurrent events of IS and factors predicting the recurrence of IS were quantified and determined using NONMEM version 7.5 software and Perl-speaks-NONMEM (PsN) version 4.1.0. After the index IS, the event was described as having recurrent IS events. All event times were treated as exact time models, in which the event was assumed to occur at the time of observation. For the baseline hazard model, three models, namely, exponential, Gompertz, and Weibull, were investigated.

### Model development

The model was developed in the following two steps: (i) a base model without any explanatory factors and (ii) an exploration of covariates.

### Development of the base model

A parametric survival function based on Equation 1 was used to describe the time to the recurrent IS.


(1) $$\begin{array}{l} S\left(t\right)=e^{-\int_{0}^{t}h\left(t\right)dt}, \end{array}$$

where *S*(*t*) is the survivor function calculated from the integral of hazard concerning time. The hazard is *h*(*t*), and the survival *S*(*t*) is a function of the cumulative hazard within the time interval between the time zero and the time *t*, describing the probability of not experiencing any recurrent IS within this interval.

The base model was developed by exploring different functions for the hazard *h*(*t*), starting from a simple time-independent constant hazard and then gradually progressing to more complex functions, including Gompertz and Weibull, according to Equations (2), (3), and (4), respectively (28).


(2) $$\begin{array}{lll} h & = & h_{0}\;\times \;e^{0} \end{array}$$

(3) $$\begin{array}{lll} h\left(t\right) & = & h_{0}\;\times \;e^{\beta t} \end{array}$$

(4) $$\begin{array}{lll} h\left(t\right) & = & h_{0}\;\times \;e^{\beta \ln\left(t\right)} \end{array}$$

The hazard of recurrent IS at baseline or baseline hazard function at different time points after the index was quantified based on Equation 5. Equation 5 shows an example of changes in the baseline hazard *h*_0_(*t*) based on different time *t* intervals.


(5) $$h_{0}(t)=\{\begin{array}{l} \;\theta_{1,\;if\;0\;<\;t<\;t\;1\;}\\ \;\theta_{2,\;}\;if\;t1<t\leq t2\\ \;\ldots \ldots \ldots \ldots \ldots \\ \;\theta_{n\;t},\;if\;t(n-1)<t\leq tn \end{array}$$

Between-subject variability around the hazard was estimated, assuming an exponential distribution for the random effect.

### Development of the covariate model

Possible explanatory variables that may influence or predict the changes in hazard were explored by including each explanatory variable in the hazard function. A parameter, β_*n*_, for each of the *n* explanatory variables, *X*_*n*_, was estimated using the following equation.


$$\begin{array}{l} h\left(t\right)=h_{0\;}\left(t\right)*\exp^{\beta 1X1+\beta 2X2\ldots .+\beta nXn}, \end{array}$$

where *h*_0_ is the baseline hazard and β_*n*_ is the coefficient for the explanatory variable, *Xn*, which describes how the hazard varies with the explanatory variable. Exponentiation of the explanatory variable coefficient provides the hazard ratio (HR), which reflects the influence of the explanatory variables relative to the hazard when the explanatory variable is not present.

Initially, the covariates were tested in a univariate manner, i.e., each covariate relationship was evaluated on the base hazard individually. Then, based on the results, covariate relationships were identified for a systematic covariate search by applying a stepwise analysis approach, i.e., with stepwise forward inclusion followed by backward elimination (29).

In the forward inclusion, the statistical significance level was set at a *P*-value of <0.05, which corresponds to a reduction of the OFV of at least 3.84, for one degree of freedom (addition of one covariate parameter). While in the backward deletion, the significant value was set to a *P*-value of <0.01, corresponding to an increase in the OFV of at least 6.64 to be kept in the model for one degree of freedom.

### Model evaluation

Parameters were estimated using the LAPLACE method (ADVAN = 6 TOL = 9 NSIG = 3) in NONMEM to obtain maximum likelihood estimates of time-to-event parameters. The parametric time-to-event (TTE) analysis was performed using NONMEM version 7.5 and Perl-speaks-NONMEM (PsN) version 4.1.0.7. Model selection was based on comparing the OFV between models, bootstrap confidence intervals for parameter estimates, and biological plausibility. The improvement in the fit was measured by a decrease (30) in the OFV generated by NONMEM. The difference in OFV between the two hierarchical models is approximately *X*^2^ distributed and can be tested for significance with *X*_1,0.052_ = 3.84.

To evaluate the predictive performance of the model throughout the model building, Kaplan-Meier visual predictive checks (VPCs) for internal and temporal validation and Xpose4 (version 4.7.1) function (31, 32) in the RStudio software (version 1.1.456, RStudio, Inc., Boston, MA, http://www.rstudio.com/) were utilized. The plots were based on simulations of 1,000 simulated datasets. To enable simulations for time points where no clinical observations had been made, extra dummy time points were added to the dataset for all individuals until 7.37 years for the VPC simulation. The parameter certainty was evaluated through relative standard error (RSE) produced from the sampling importance resampling (SIR) method (33).

## Results

Out of 7,697 subjects, 333 patients (4.32%) developed recurrent IS within the maximum follow-up period of 7.37 years. The median time to the first recurrent IS was 1.2 years. The study population included all age groups, from young to elderly, with a median age of 63.47 years at the time of index IS. As shown in Table 1, most of the patients were women (4,289, 55.72%). The percentage of smokers in this study population was 48%. Of 7,697 subjects, 3,493 (45.38%) subjects had diabetes before index IS, while the number of patients with HTN before index IS was 5,506 (71.5%). The number of subjects with HPLD before index IS was 2,028 (26.34%), of patients who had IHD before index IS was 879 (11.4%), and of patients who had AF before index IS were 3.4%. Among patients who had recurrent IS, the percentage of patients who received antiplatelets (APLT), antihyperlipidemics, angiotensin-converting enzyme inhibitors (ACEI), beta-blockers (BB), calcium channel blockers (CCB), diuretics (DIU), and antidiabetics (ADM) for concurrent disease control and secondary prevention were 85.58%, 86.18%, 29.42%, 11.71%, 24.02%, 8.70%, and 39.63%, respectively.

**Table 1:** Characteristic of patients with recurrent IS during different time intervals that included into the study (*N* = 333).

**Variable**	**Patients with recurrent IS *N* = 333 (%)**	**Patients with no recurrent IS *N* = 7,364 (%)**
Recurrent IS < 6 months	108 (31.43)	**–**
**Age group**		
< 60	150 (45.04)	2,924 (39.70)
≥60	183 (54.95)	4,440 (60.29)
Female	186 (55.85)	4,103 (55.71)
2^nd^ recurrent stroke	36 (0.108)	–
**Ethnicity**		
Malay	155 (46.54)	1,479 (20.08)
Chinese	7 (2.10)	206 (2.79)
Indian	3 (0.9)	80 (1.08)
Others	167 (50.15)	5,601 (76.05)
Smoker	202 (60.66)	3,547 (48.166)
DM	195 (58.55)	3,298 (44.78)
**Duration of diabetes (years)**		
< 1	9 (2.70)	340 (4.61)
1–5	97 (29.12)	1,675 (22.74)
6–10	42 (12.61)	520 (7.06)
>10	43 (12.91)	763 (10.36)
Unknown	4 (1.2)	–
Family history of stroke	27 (8.10)	339 (4.60)
HTN	288 (86.48)	5,218 (70.85)
**HTN duration (years)**		
≤ 5	163 (48.94)	2,167 (29.42)
>5	125 (37.53)	2,051 (27.85)
IHD	77 (23.12)	802 (10.89)
HPLD	159 (47.74)	1,869 (25.38)
AF	4 (1.2)	263 (3.57)
HU	16 (4.8)	218 (2.96)
**NIHSS**		
Minor	145 (43.54%)	3,407 (46.26%)
Moderate/severe	188 (56.45%)	3,957 (53.73%)
**Received medications for concurrent disease control and/or secondary prevention**		
APLT	285 (85.58%)	6,613 (89.80%)
Antihyperlipidemic	287 (86.18%)	6,607 (89.72%)
ACEI	98 (29.42%)	2,298 (31.20%)
BB	39 (11.71%)	776 (10.53%)
CCB	80 (24.02%)	1,520 (20.64%)
DIU	29 (8.70%)	425 (5.77%)
ADM	132 (39.63)	2,306 (31.31%)

ACEI, angiotensin converting enzyme inhibitors; ADM, antidiabetics; AF, atrial fibrillation; APLT, antiplatelet; BB, beta blockers; CCB, calcium channel blockers; DIU, diuretics; DM, diabetes mellitus; FHOS, family history of stroke; IHD, ischemic heart disease; HTN, hypertension; HPLD, hyperlipidemia; HU, hyperuricemia; NIHSS, national institute of health stroke scale; N, number of patients.

### Baseline hazard model of recurrent IS

**Figure 1:**
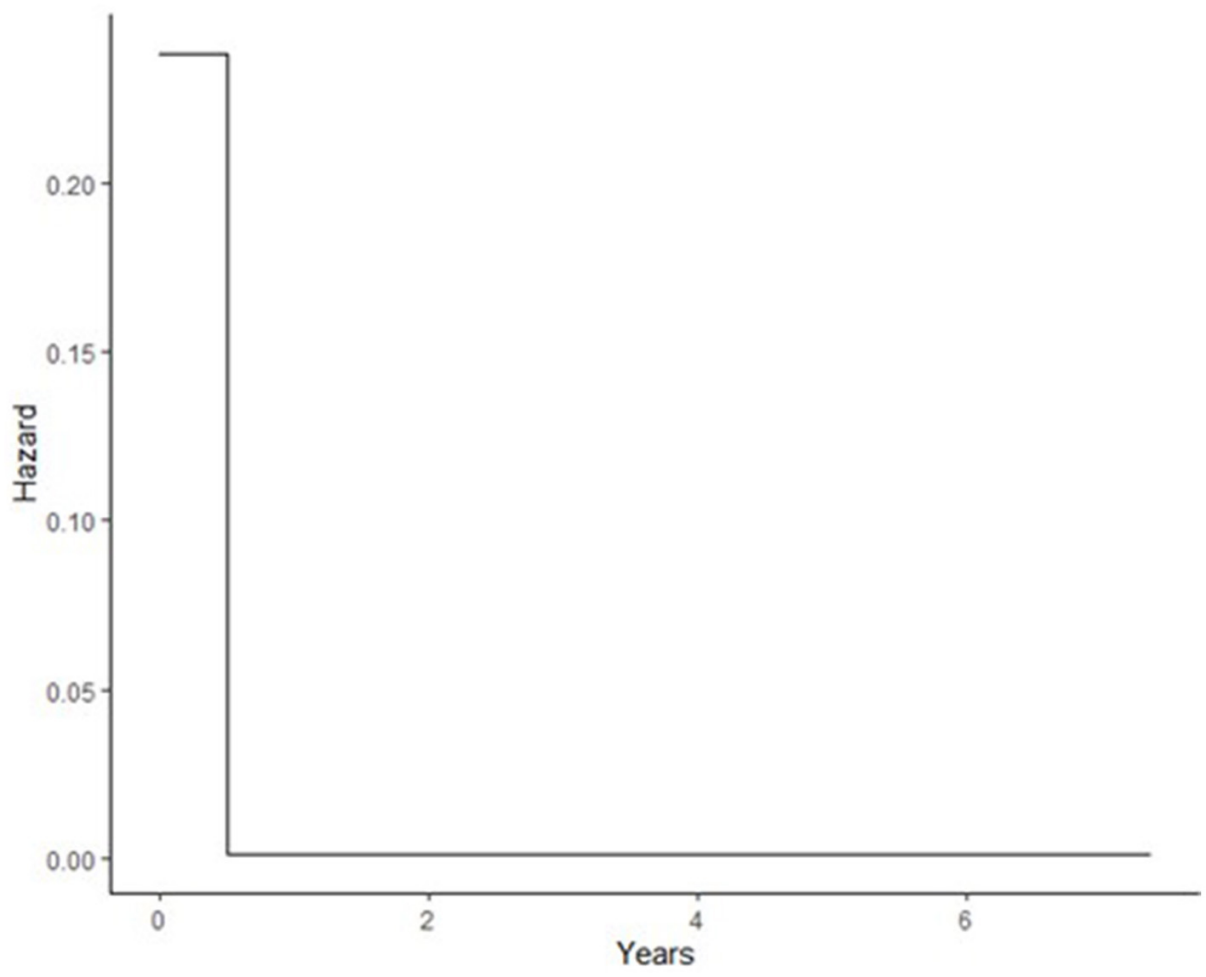
Baseline hazard during different time intervals after index IS; during first 6 months after index IS, after 6 months.

The Gompertz model fits the data well in terms of OFV, clinical plausibility, and the Kaplan–Meier plots. The baseline hazard of recurrent IS was quantified at two different time points, as shown in Table 2. As shown in Table 3, the hazard of recurrent IS when the predictor variables were set to zero was 0.238 in the first 6 months after the index IS, and the hazard remained non-zero afterward (Figure 1). After incorporating the factor of time and established risk factor, the exponential increase in the hazard of recurrent IS was observed in the first 3 years after the index IS and then exponentially reduced afterward (**Figure 3**).

**Table 2:** Objective function value differences between different models.

**Number of parameters**	**Variable**	**Model**	**OFV**	**ΔOFV**	***p*-value**
1	Constant	*h*(*t*) = θ_1_	3,803.971	0	–
2	Gompertz	$h\left(t\right)=\theta_{1}\;\times \;e^{\left(\theta_{2}\right)t}$	3,777.083	−26.888	< 0.0001^*^
2	Weibull	$h\left(t\right)=\theta_{1}\;\times \;e^{\left(\theta_{2}\right)\ln\left(t\right)}$	3,788.58	−15.391	0.003955^*^
**After inserting different time intervals**					
4	Gompertz	$h\left(t\right)=\theta_{x}\;\times \;e^{\left(\theta_{y}\right)t}$	2,808.68	−959.291	< 0.0001^*^
4	Weibull	$h\left(t\right)=\theta_{x}\;\times \;e^{\left(\theta_{y}\right)\ln\left(t\right)}$	3,185.810	−623.161	< 0.0001^*^

OFV, objective function value; h, hazard; t, time; θ_*x*_ equals, θ_1_ if time < 0.5 year; θ_3_ if time ≥ 0.5, θ_*y*_ equals; θ_2_ if time < 3 years, θ_4_ if time ≥ 3 years. ^*^Significance; p-value < 0.05.

**Table 3:** Parameters of the final developed model for recurrent IS after index IS.

**Parameter**		**Description**	**Typical value**	**Half-life (Ln2/α)**	**aHR 95%CI**	**RSE%**
θ_1_(< 6 months)	θ_1_	Baseline hazard	0.238	–		19.92%
θ_3_(≥6 months)	θ_3_		0.0016			21.62%
α (< 3)	θ_2_	Shape parameter in the first 3 years after index IS	1.63	0.42 (5.06 months)		4.81%
α (≥3)	θ_4_	Shape parameter after 3 years of index IS	0.23	3.008 years		20.19%
HPLD (covariate)	θ_5_	Effect of baseline HPLD on hazard	0.799	–	2.22 (1.81–2.72)	12.89%
IHD (covariate)	θ_6_	Effect of baseline IHD on hazard	0.745	–	2.10 (1.64–2.69)	16.85%
HTN (covariate)	θ_7_	Effect of baseline HTN on hazard	0.711	–	2.03 (1.52–2.71)	20.62%
APLT	θ_8_	Effect of receiving APLT on hazard	−0.514		0.59 (0.79–0.44)	28.41%

APLT, antiplatelet; h, baseline hazard; RSE, relative standard error; 95% CI, 95% confidence interval; α, shape parameter; aHR, adjusted hazard ratio; HPLD, hyperlipidaemia; HTN, hypertension; IHD, ischemic heart disease; NIHSS, national institute of heath stroke scale; The RSE (%) were obtained from sampling importance resampling (SIR) method (33).

### Factors influencing the risk of having recurrent IS after index IS

**Figure 2:**
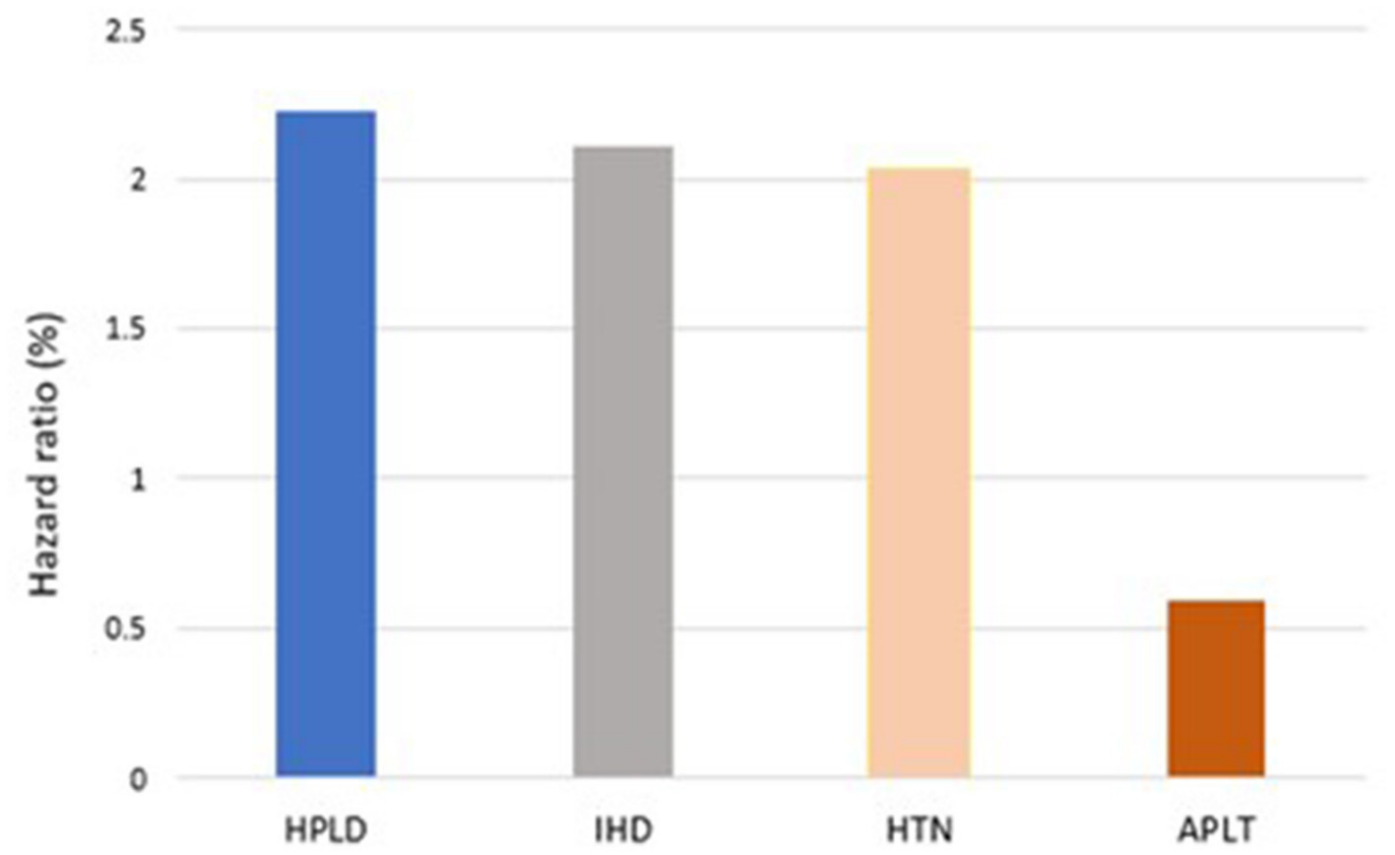
Effect of covariates on hazard of recurrent IS after index IS.

**Figure 3:**
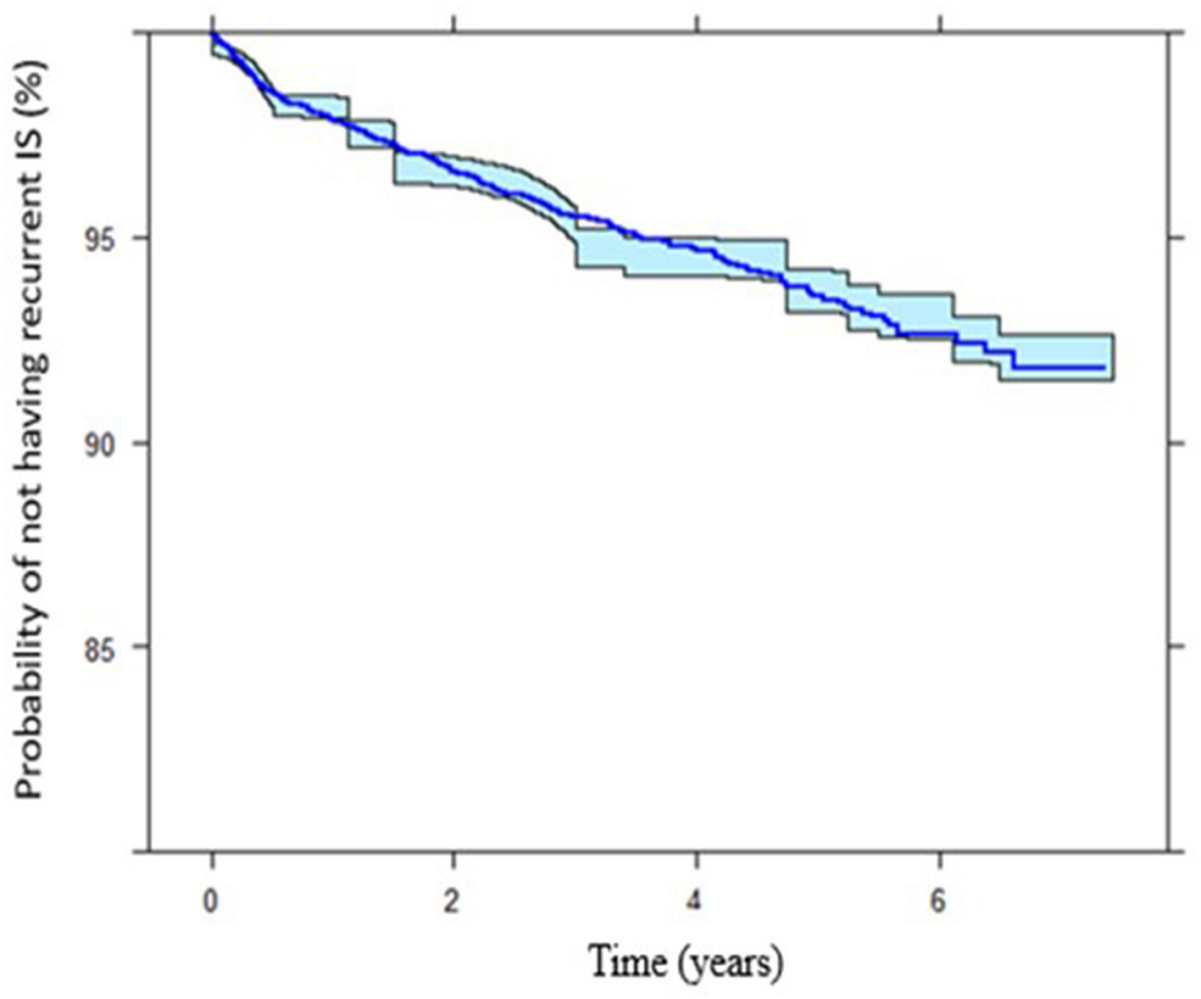
Kaplan-Meier plots showing the IS survivor function (probability of not having recurrent ischemic stroke) throughout different time intervals. The final time-to-event model of the internal data.

In our model, the presence of established cardiovascular risk factors prior to index IS determine the risk of recurrent IS. Prior to index IS, diagnosis of HPLD, HTN, and IHD increases the risk of recurrent IS with [HR, 2.22 (95%CI: 1.81–2.72)], [HR, 2.03 (95%CI: 1.52–2.71)], and [HR, 2.10 (95%CI: 1.64–2.69)], respectively, while receiving APLT for secondary prevention decreased this hazard [HR, 0.59 (95%CI: 0.79–0.44)] (Figure 2). The Kaplan-Meier VPCs for recurrent IS after index IS showed good predictions (Figure 3).

**Figure 4:**
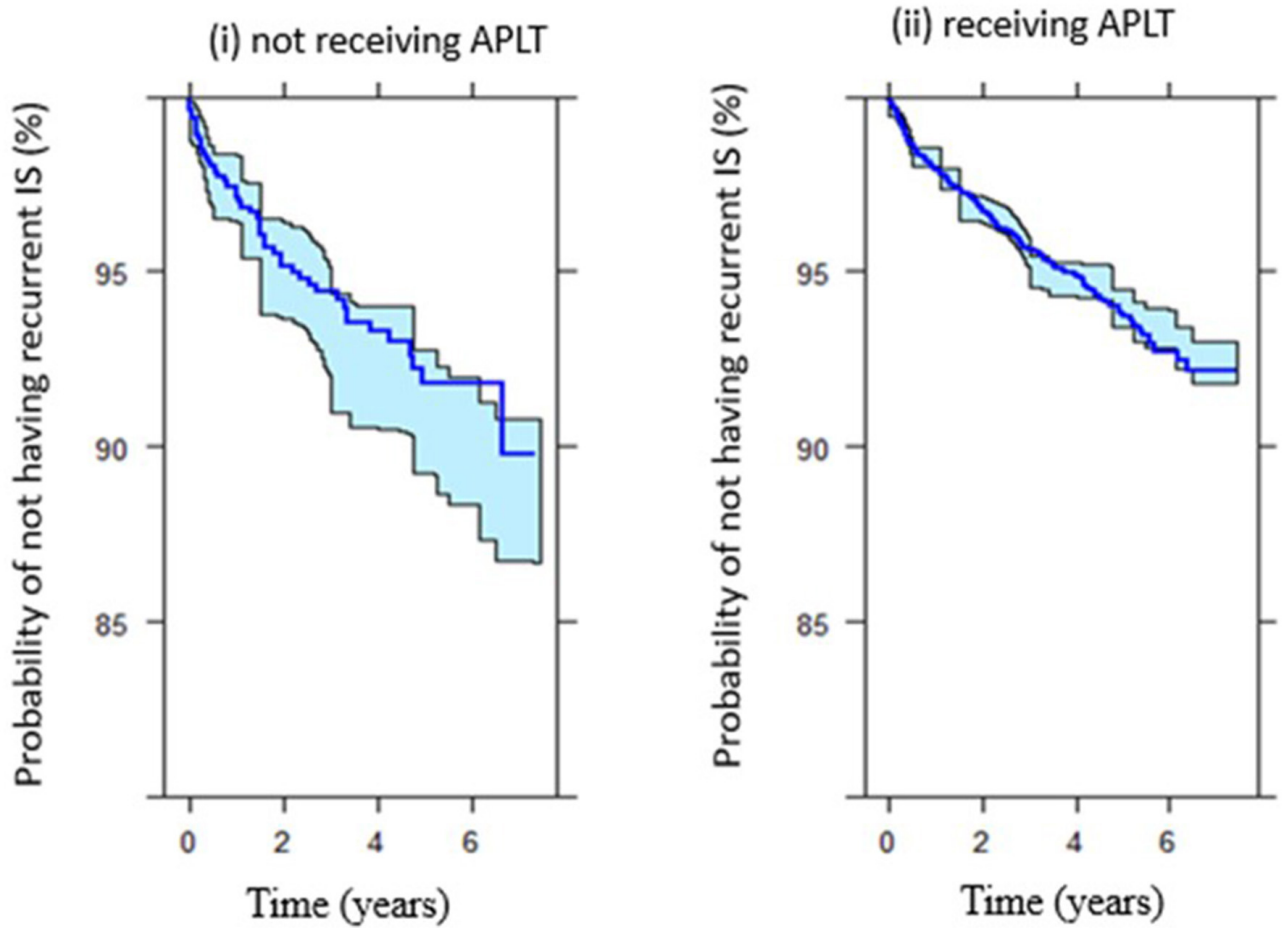
Survival (probability of not having recurrent IS after index IS) among **(i)** patients did not receive APLT vs. **(ii)** patients received APLT.

Figure 4 shows the survival (probability of not having recurrent IS) among patients who received APLT vs. patients who did not receive APLT for secondary prevention.

## Discussion

To the best of our knowledge, this is the first study predicting the recurrence of IS in our population using real-world data of IS population as well as defining the baseline hazard of recurrent IS. A previous study (34) reported a constant hazard of having recurrent IS over time. In our population, the hazard of recurrent IS was reported to change over time after the first IS attack. Unlike the conventional model development (e.g., the Cox model), defining the specific shape or distribution of the event hazard at baseline (e.g., just after the index stroke) may allow for better prediction of the event of interest, taking into account the “natural effect” of the disease itself.

Recurrent stroke is associated with increased disability and mortality rates compared to index stroke (35). Even with appropriate secondary prevention, the risk of recurrence after IS is high, especially in the early phase after stroke (36). It has been reported that, within the first year after the initial stroke, the risk of stroke recurrence is higher (between 6 and 14%) as opposed to the risk in subsequent years (4% annually) (37–39). A more recent study showed that the incidence of stroke recurrence was the highest during the first year after index stroke at 12.8% with a declining annual rate, 6.3% during the second year, and 5.1% (95% CI, 4.0–6.5) during the third year after the index stroke (15).

In our population, we demonstrated the predicted hazard of recurrent IS at certain time points and change over time. Those with ≥2 concomitant diseases predicted a higher likelihood (>3.5%) of recurrent IS as compared to those who had at least one or no risk factor. This indicates that early and extensive secondary IS prophylaxis, especially in the first 3 years after the index IS as well as for those with the three risk factors, is paramount to prevent recurrent IS. The follow-up schedule after the index IS should be personalized depending on the risk factors. Those with more risk factors may require frequent follow-up after the stroke as well as different therapy goals for controlling the concomitant diseases.

The time course of recurrent IS hazards may represent the infarct involved during the stroke attack. There may be a relatively rapid increase in infarction cells after the initial diagnosis of stroke, which may increase the hazard of recurrent IS during the stage. However, the incidence of recurrent IS observed in the surviving population may decrease with time. This could be explained by the fact that the secondary prophylaxis therapy received may show a delay in obvious benefit in reducing the recurrent IS at this stage but with greater benefit later.

In this study, IHD, HPLD, and HTN were identified as independent predictors for recurrent IS. These findings are consistent with data reported in a previous study (22–25). The presence of HPLD, IHD, or HTN was found to increase the hazard of developing recurrent IS by 2.22, 2.10, and 2.03, respectively. In contrast, receiving APLT was found to decrease the hazard of recurrent IS by ~40%.

HPLD findings could be explained through the angiopathy resulting from atherosclerotic plaque (40). For IHD, it was reported that IHD and IS share similar pathophysiology, mainly because atherosclerosis is manifested in both conditions (22). Patients who have atherosclerosis are at risk for acute stroke. In both cases, a sudden change in circulation arises, and as a result, the blood supply decreases to some parts of the brain or heart (22). In agreement with these findings, receiving APLT was found to decrease the hazard of recurrent IS among the whole population with index IS. Effective management of these comorbidities is necessary to reduce the risk of recurrent IS. Although we reported the established and well-known risk factors of recurrent IS, our model allows the prediction and quantification of the recurrent IS hazard at different time points after the index IS. Moreover, the hazard is quantified according to the risk groups, which allows the future study to incorporate the time-varying effect of secondary prophylaxis therapies on the progression and hazard of recurrent IS.

## Limitations

This was a retrospective study based on the available data from the National Stroke Registry of Malaysia. Therefore, the first stroke captured by the NNEUR from 2009 to 2016 was assumed to be the first stroke experienced by the patient. Any data on the prior TIA or stroke before the NNEUR establishment were not available and not considered in the current study. Due to the nature of the data captured from the registry database, the comorbidities were analyzed independently. Nevertheless, this study was a population-based study and large samples representing various ethnic groups across the country. This model may provide insights into the importance of frequent follow-up, especially in the early days (examples within the first 6 months to 1 year), and thus perhaps may make a positive shift in the Malaysian population regarding follow-up schedules during the management to prevent recurrent IS. This model is expected to be the basic model for future studies incorporating the time-varying effects of drugs, e.g., dosing changes and pharmacokinetic and pharmacodynamic characteristics.

## Conclusion

Incorporating time in predicting the risk of recurrent IS may attribute positively to predicting the prognosis of recurrent IS. The hazard of recurrent IS changes over time after the index IS. In addition to concomitant diseases, secondary prevention time also plays a vital role in predicting the risk of recurrent IS population. These results may add to the knowledge related to patient follow-up schedules during the management of IS to prevent IS recurrence.

## Data availability statement

The raw data supporting the conclusions of this article will be made available by the authors, upon request.

## Ethics statement

The studies involving human participants were reviewed and approved by Medical Research and Ethics Committee (MREC), Ministry of Health, Malaysia (Research ID: NMRR-08-1631-3189). The patients/participants provided their written informed consent to participate in this study. Written informed consent was obtained from the individual(s) for the publication of any potentially identifiable images or data included in this article.

## Author contributions

Conception and design: ME, SNH, SMSG, and OA. Data acquisition: IL, NNS, and ZAA. Drafting the manuscript: ME and SNH. Critically revising and reviewing the submitted version of the manuscript: All authors. All authors read and approved the final manuscript.
