# Supplementary material for: Predictive model of recurrent ischemic stroke: model development from real-world data
Source: Front Neurol. 2023 Apr 28;14:1118711. doi: 10.3389/fneur.2023.1118711 (PMC10176964; doi:10.3389/fneur.2023.1118711)
Supplement: Supplementary file 1 [file Table_1.docx]

**Table1_S** Univariate and multivariate analysis of covariate effects on the hazard of recurrent IS after index IS (n = 333)

| **Parameter** | | **Model** | **∆OFV** | ***p-*Value** |
| --- | --- | --- | --- | --- |
| Univariate covariate testing | | | | |
| Base, no covariate | $h(t)=\theta_{x} \times e^{(\theta_{y})t}$  OFV=2808.68 | | 0 | - |
| HPLD | $h\left( t \right)=\theta_{x} \times e^{(\left( \theta_{y} \right)t+\theta5(HPLD))}$ | | -71.06 | <0.0001 |
| IHD | $h\left( t \right)=\theta_{x} \times e^{(\left( \theta_{y} \right)t+\theta5(IHD))}$ | | -49.02 | <0.0001 |
| HTN | $h\left( t \right)=\theta_{x} \times e^{(\left( \theta_{y} \right)t+\theta5(HTN))}$ | | -44.02 | <0.0001 |
| DM | $h\left( t \right)=\theta_{x} \times e^{(\left( \theta_{y} \right)t+\theta5(DM))}$ | | -21.75 | <0.0001 |
| APLT | $h\left( t \right)=\theta_{x} \times e^{(\left( \theta_{y} \right)t+\theta5(APLT))}$ | | -10.09 | 0.0014 |
| ADM | $h\left( t \right)=\theta_{x} \times e^{(\left( \theta_{y} \right)t+\theta5(ADM))}$ | | -5.39 | 0.0202 |
| HU | $h\left( t \right)=\theta_{x} \times e^{(\left( \theta_{y} \right)t+\theta5(HU))}$ | | -4.65 | 0.031 |
| BB | $h\left( t \right)=\theta_{x} \times e^{(\left( \theta_{y} \right)t+\theta5(BB))}$ | | -2.05 | 0.152 |
| DIU | $h\left( t \right)=\theta_{x} \times e^{(\left( \theta_{y} \right)t+\theta5(DIU))}$ | | -2.87 | 0.09 |
| AF** | $h\left( t \right)=\theta_{x} \times e^{(\left( \theta_{y} \right)t+\theta5(AF))}$ | | -0.44 | 0.507 |
| FHOS** | $h\left( t \right)=\theta_{x} \times e^{(\left( \theta_{y} \right)t+\theta5(FHOS))}$ | | -2.735 | 0.09 |
| Gender** | $h\left( t \right)=\theta_{x} \times e^{(\left( \theta_{y} \right)t+\theta5(Gender))}$ | | -0.435 | 0.509 |
| ACEI | $h\left( t \right)=\theta_{x} \times e^{(\left( \theta_{y} \right)t+\theta5(ACEI))}$ | | -0.03 | 0.862 |
| CCB | $h\left( t \right)=\theta_{x} \times e^{(\left( \theta_{y} \right)t+\theta5(CCB))}$ | | -1.52 | 0.217 |
| NIHSS | $h\left( t \right)=\theta_{x} \times e^{(\left( \theta_{y} \right)t+\theta5(NIHSS))}$ | | -1.103 | 0.293 |
| **Forward inclusion of covariates** | | | | |
| Base Model | $h\left( t \right)=\theta_{x} \times e^{(\left( \theta_{y} \right)t+\theta5(HPLD))}$  OFV=2737.615 | | 0 | - |
| IHD | $h\left( t \right)=\theta_{X} \times e^{\left( \left( \theta_{y} \right)t+\theta5\left( HPLD \right)+\theta6\left( IHD \right) \right)}$ | | -38.516 | <0.0001 |
| HTN | $h\left( t \right)=\theta_{X} \times e^{\left( \left( \theta_{y} \right)t+\theta5\left( HPLD \right)+\theta6\left( IHD \right)+\theta7\left( HTN \right) \right)}$ | | -26.14 | <0.0001 |
| DM | $h\left( t \right)=\theta_{X} \times e^{\left( \left( \theta_{y} \right)t+\theta5\left( HPLD \right)+\theta6\left( IHD \right)+\theta7\left( HTN \right)+\theta8\left( DM \right) \right)}$ | | -3.96 | 0.046 |
| APLT | $h\left( t \right)=\theta_{X} \times e^{\left( \left( \theta_{y} \right)t+\theta5\left( HPLD \right)+\theta6\left( IHD \right)+\theta7\left( HTN \right)+\theta8\left( DM \right)++\theta9\left( APLT \right) \right)}$ | | -10.39 | 0.0012 |
| ADM | $h\left( t \right)=\theta_{X} \times e^{\left( \left( \theta_{y} \right)t+\theta5\left( HPLD \right)+\theta6\left( IHD \right)+\theta7\left( HTN \right)+\theta8\left( DM \right)++\theta9\left( APLT \right)+\theta10\left( ADM \right) \right)}$ | | -0.167 | 0.682 |
| HU | $h\left( t \right)=\theta_{X} \times e^{\left( \left( \theta_{y} \right)t+\theta5\left( HPLD \right)+\theta6\left( IHD \right)+\theta7\left( HTN \right)+\theta8\left( DM \right)++\theta9\left( APLT \right)+\theta10\left( HU \right) \right)}$ | | -1.055 | 0.304 |
| **Backward elimination of covariates** | | | | |
|  | $h\left( t \right)=\theta_{X} \times e^{\left( \left( \theta_{y} \right)t+\theta5\left( HPLD \right)+\theta6\left( IHD \right)+\theta7\left( HTN \right)+\theta8\left( DM \right)++\theta9\left( APLT \right) \right)}$ | |  |  |
| APLT | $h\left( t \right)=\theta_{X} \times e^{\left( \left( \theta_{y} \right)t+\theta5\left( HPLD \right)+\theta6\left( IHD \right)+\theta7\left( HTN \right)+\theta8\left( DM \right) \right)}$ | | +10.93 | 0.0009 |
| DM | $h\left( t \right)=\theta_{X} \times e^{\left( \left( \theta_{y} \right)t+\theta5\left( HPLD \right)+\theta6\left( IHD \right)+\theta7\left( HTN \right)+\theta8\left( APLT \right) \right)}$ | | +3.88 | 0.048 |
| HTN | $h\left( t \right)=\theta_{X} \times e^{\left( \left( \theta_{y} \right)t+\theta5\left( HPLD \right)+\theta6\left( IHD \right)+\theta7\left( APLT \right) \right)}$ | | +24.67 | <0.0001 |
| IHD | $h\left( t \right)=\theta_{X} \times e^{\left( \left( \theta_{y} \right)t+\theta5\left( HPLD \right)+\theta6\left( HTN \right)+\theta7\left( APLT \right) \right)}$ | | +32.291 | <0.0001 |
| HPLD | $h\left( t \right)=\theta_{X} \times e^{\left( \left( \theta_{y} \right)t+\theta5\left( IHD \right)+\theta6\left( HTN \right)+\theta7\left( APLT \right) \right)}$ | | +55.19 | <0.0001 |
| Final developed model | $h\left( t \right)=\theta_{X} \times e^{\left( \left( \theta_{y} \right)t+\theta5\left( HPLD \right)+\theta6\left( IHD \right)+\theta7\left( HTN \right)+\theta8\left( APLT \right) \right)}$ | |  |  |

*Abbreviations: ACEI; angiotensin converting enzyme inhibitors, ADM; antidiabetics, AF; atrial fibrillation, APLT; antiplatelet, BB; beta blockers, CCB; calcium channel blockers, DIU; diuretics, DM: Diabetes mellitus, FHOS; family history of stroke, IHD; Ischemic heart disease, HTN; Hypertension, HPLD; Hyperlipidemia, HU; hyperuricemia, NIHSS; National Institute of Health Stroke scale, N; Number of patients, ∆OFV is the difference in objective function value between covariate and base model: OFVCovariate – OFVBase. . h; hazard, t;* $\theta_{x}$ *equals;* $\theta_{1}$*if time < 0.5 year,* $\theta_{3}$ *if time ≥ 0.5,* $\theta_{y}$ *equals;* $\theta_{2}$*if time < 3 years,* $\theta_{4}$ *if time ≥ 3 years. †The potential covariate was included in the stepwise forward inclusion then backward elimination time. * Significance; p value < 0.05 in univariate analysis and stepwise forward inclusion. Significance <0.01 in backward elimination.*

** Highlighted in blue were the significant variables.*
